# Exploiting nonlinear properties of pure and Sn-doped Bi_2_Te_2_Se for passive Q-switching of all-polarization maintaining ytterbium- and erbium-doped fiber lasers

**DOI:** 10.1038/s41598-017-07706-7

**Published:** 2017-08-07

**Authors:** Jakub Bogusławski, Maciej Kowalczyk, Przemysław Iwanowski, Andrzej Hruban, Ryszard Diduszko, Kazimierz Piotrowski, Krzysztof Dybko, Tomasz Wojciechowski, Marta Aleszkiewicz, Jarosław Sotor

**Affiliations:** 1 Laser & Fiber Electronics Group, Faculty of Electronics, Wrocław University of Science and Technology, Wybrzeże S. Wyspiańskiego 27, 50-370 Wrocław, Poland; 2 Institute of Physics, Polish Academy of Sciences, Aleja Lotnikow 32/46, PL-02-668 Warsaw, Poland; 3 International Research Centre MagTop, Aleja Lotników 32/46, PL-02-668 Warsaw, Poland; 4 Tele and Radio Research Institute, Ratuszowa 11, PL-03-450 Warsaw, Poland

## Abstract

Due to their broadband nonlinear optical properties, low-dimensional materials are widely used for pulse generation in fiber and solid-state lasers. Here we demonstrate novel materials, Bi_2_Te_2_Se (BTS) and Sn-doped Bi_2_Te_2_Se (BSTS), which can be used as a universal saturable absorbers for distinct spectral regimes. The material was mechanically exfoliated from a bulk single-crystal and deposited onto a side-polished fiber. We have performed characterization of the fabricated devices and employed them in polarization-maintaining ytterbium- and erbium-doped fiber lasers. This enabled us to obtain self-starting passively Q-switched regime at 1 µm and 1.56 µm. The oscillators emitted stable, linearly polarized radiation with the highest single pulse energy approaching 692 nJ. Both lasers are characterized by the best performance observed in all-polarization maintaining Q-switched fiber lasers with recently investigated new saturable absorbers, which was enabled by a very high damage threshold of the devices. This demonstrates the great potential of the investigated materials for the ultrafast photonics community.

## Introduction

The 2016 Nobel Prize in Physics was awarded for theoretical works which predicted a new topological state of matter. Novel symmetry protected topological phases were identified, while investigating two-dimensional systems. These findings were subsequently verified in experiments discovering both two and three dimensional topological insulators^1^. This state of matter is characterized by insulating or semi-conducting bulk states and graphene-like gapless states at the surface.

According to famous R. Feynman quote, “*physics is like sex*” and also these results does not necessarily have to induce potential applications to be extremely valuable for the scientific community. However, as in most cases of groundbreaking discoveries – they do, and topological insulators can be potentially exploited in the field of spintronics or quantum computing^2, 3^. At the same time, the great interest in those materials paved a way to a variety of optoelectronic applications^4^, which in contrary to previously mentioned, has already been experimentally implemented. Those include the use in optical switching^5^, second harmonic generation^6^, plasmonics^7^ and spatial self-phase modulation^8^. One of the extraordinary properties of topological insulators is their strong optical nonlinearity manifesting in saturable absorption. At first it was demonstrated in Bi_2_Te_3_ in the near infrared^9^, however it was promptly shown that saturable absorption exists in extremely broad spectral range due to the presence of surface states^10^. Consequently, such materials can be employed as a passive optical switch for Q-switching or mode-locking of fiber^9^, solid state^11^ and waveguide lasers^12^. It was soon demonstrated in various spectral regions of near- and mid-infrared^13, 14^. Saturable absorption was rapidly demonstrated in similar materials, like Bi_2_Se_3_^15, 16^ or Sb_2_Te_3_^17, 18^.

Large energy pulsed lasers have become indispensable in many areas, like material processing or triggering of nonlinear optical processes. The main issue in generation of large energy pulses in passively Q-switched fiber lasers (PQS) is the damage threshold of the saturable absorber. In order to increase its value it is possible to deposit the absorbing material on a surface of a side-polished fiber. In this configuration one can exploit interaction between the evanescent field propagating in the fiber cladding and the material. Since the beam is not directly incident on the sample, the intensity of the interacting field is relatively low – this significantly increases a damage threshold (in regard to intracavity laser power). The magnitude of the nonlinear interaction can be controlled by change of the deposition length, geometrical dimensions of the fiber or the deposition of an overlay^19^. Another possibility is to control the optical properties of a material by electrical gating exploiting the electro-optical effect in e.g. graphene^20^. A number of results with low-dimensional materials in this configuration were reported in the literature, including carbon nanotubes^21^, graphene^22^, graphene oxide^23^, transition metal dichalcogenides^24^ (e.g. MoS_2_), black phosphorus^25, 26^, bismuth telluride^27^ and antimony telluride^28^. However, mentioned experiments cover mostly non-polarization maintaining laser architectures. Such devices are vulnerable to external factors (e.g. vibrations, temperature changes) which may interrupt their proper operation. Thereupon, lasers employed for industrial applications should be polarization maintaining as they may be operated in harsh environment.

All of the above examples based on topological insulators were based on binary materials, like Bi_2_Te_3_, Bi_2_Se_3_ and Sb_2_Te_3_. Ternary topological insulators, like Bi_2_Te_2_Se (BTS), attributed a considerable attention due to the simple structure with one Dirac cone crossing the band gap, which makes it attractive for experimental investigations^29^. It has a well ordered structure comprising of Te-Bi-Se-Bi-Te quintuple layers. Stoichiometric BTS is always a heavy doped n-type material showing metallic behavior. Higher bulk resistivities with the lowest possible bulk concentration can be obtained by applying the composition process. The most effective way is by introducing light Sn doping of stoichiometric melt^30, 31^. The as obtained crystal is p-type high sensitive at low temperature with carrier density as low as 8 × 10^4^ cm^−3^. This crystal displayed the highest resistivity ($\sim$ 10 ohm $\bullet$ cm) and the lowest carrier concentration due to the special nature of Sn impurity in BTS – Sn dopants act as a carrier concentration “buffer” absorbing electrons or holes into localized band. Additionally, Sn impurity influences the electronic band structure of the material, creating a defect states close to the top of the valence band. In consequence, p-type Sn-doping increases the contribution of the surface conduction states typical for topological insulators.

It is interesting to see what kind of optical properties and phenomena those materials may offer. Ternary TIs has already been recognized to have a broadband and wavelength-independent saturable absorption^32, 33^. Wang *et al*. has determined nonlinear optical properties of Bi_2_Te_*3-x*_Se_*x*_ two-dimensional nanosheets, showing that it strongly depends on the atomic structure of the compound, being the strongest in Bi_2_Te_2_Se. In this material both bulk and the surface states can participate in the saturable absorption in near infrared and the authors attributed the variations of the measured parameters to the different contributions of these two^32^. Nevertheless, until now there was no demonstrations of practical application of those materials nor investigations of optical properties of doped materials. Moreover, it is questionable if the nonlinear optical phenomena in such materials originate primarily from the bulk or surface topological states.

Here we demonstrate the fabrication of fiber integrated saturable absorber devices based on BTS and BSTS. The layer of each material was mechanically exfoliated and deposited onto a surface of a side-polished fiber. We have studied the nonlinear absorption properties of both devices observing that they are characterized by high modulation depth and damage threshold exceeding 1 μJ. No significant influence of Sn doping on saturable absorber’s parameters was observed at near-infrared wavelengths. Subsequently, both materials have been employed for passive Q-switching of fiber lasers operating at 1 µm and 1.56 µm spectral ranges, based on ytterbium- and erbium-doped active fibers. Both lasers were characterized by exceptional operation characteristics in terms of average power, pulse energy and duration. Similar performance was noted with BTS and BSTS saturable absorbers. The cavity comprises solely polarization maintaining fibers and components, hence the emitted radiation is linearly polarized. Moreover, the lasers were insensible to various disturbing factors, like mechanical vibrations. The ytterbium-doped fiber laser with BSTS-based saturable absorber was generating stable pulse trains around 1030 nm with the highest single pulse energy of 356 nJ without damaging the absorber. The repetition frequency could be tuned up to 108 kHz and the shortest pulse duration amounted to 960 ns. The highest energy obtained in erbium-doped fiber laser was 692 nJ with repetition rate tunable from 64 kHz up to 205 kHz and minimal pulse duration of 520 ns. Both lasers are characterized by the best performance observed in all-polarization maintaining PQS fiber lasers based on newly investigated saturable absorbers.

## Results

### Material characterization

**Figure 1:**
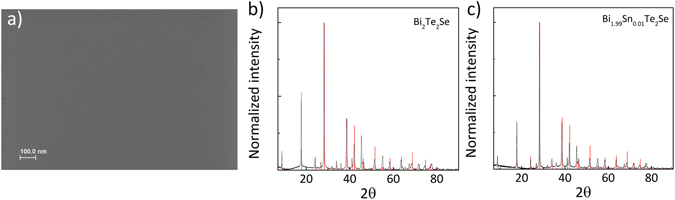
Structural characterization of obtained BTS and BSTS crystals. FESEM image of BSTS crystal surface (**a**). X-Ray powder diffraction patterns of BTS (**b**) and BSTS crystal (**c**).

**Figure 2:**
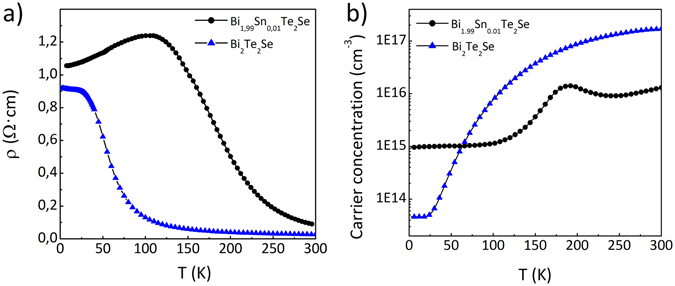
Electrical characterization of obtained BTS and BSTS crystals. Resistivity dependence on temperature of the crystals (**a**). Temperature dependence of carrier concentration of the crystals (**b**).

Field emission scanning electron microscopy (FESEM) was used to study both crystal’s surfaces. In both cases flat surface without any islands or separations was observed, which is shown in Fig. 1(a) on the example of BSTS crystal (corresponding image for pure BTS with complementing EDX spectrum can be found in Supplementary Information, see Fig. S1). The obtained X-Ray powder diffraction patterns are shown in Fig. 1(b) and (c) for BTS and BSTS, respectively. The measurement confirmed that both materials have the same, good crystal structure with no additional phases. Next, the samples were cleaved along (001) crystallographic plane for temperature dependent bulk resistivities and carrier concentration measurements. Figure 2(a) shows resistivity ρ vs. temperature dependence for 400 μm thick BTS and BSTS samples. In the first case the resistivity saturates below 50 K. Value of ρ for Sn-doped crystal strongly increase on cooling which is characteristic of an insulator and saturates below $\sim$ 12  K. This behavior corresponds to the metallic surface transport as previously described^34^. Low temperature resistivity above 1 Ohm·cm and about 1 Ohm·cm are typical for high quality BSTS and BTS crystals, respectively. Figure 2(b) shows the temperature dependence of carrier density for 170 μm thickness of both samples. Low temperature of p-type carrier density was found to be 9 × 10^14^ cm^−3^ for BSTS and 4.6 × 10^13^ cm^−3^ for BTS crystals. This shows that Sn doping increases the bulk resistivity and reduces carrier concentration. All of these measurements confirm the high quality of the measured samples and provide a basis for measuring the other properties of topological insulators.

### Fiber-integrated saturable absorbers

**Figure 3:**
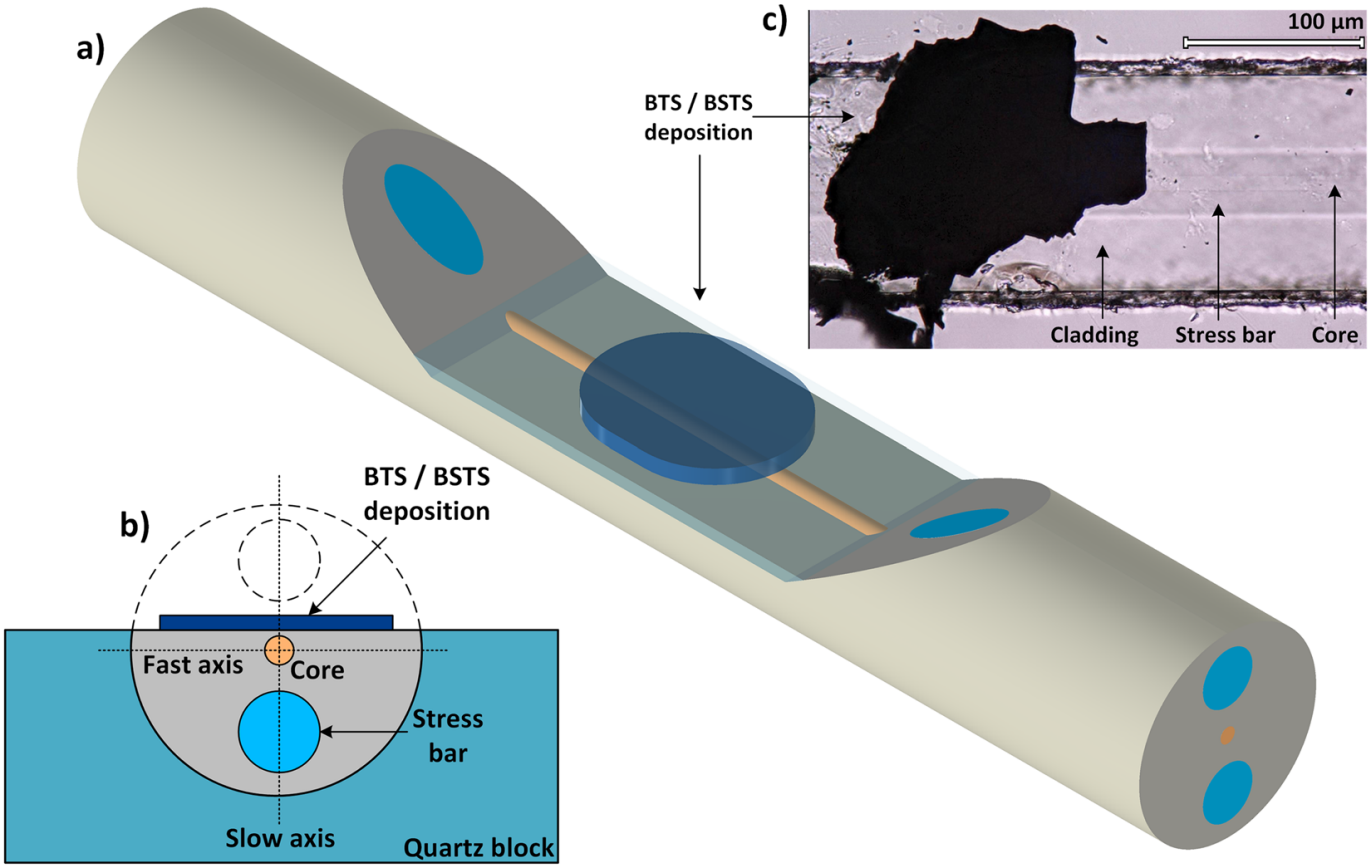
Schematic of the fabricated fiber device (not in scale). The PM fiber is polished parallel to fast axis. Distance between cladding and core along the cut is approx. 1–2 µm. Transparency of the upper part of the cladding along the cut is introduced and the quartz block is not shown in order to depict the scheme more clearly (**a**). Cross-section of the fiber along the cut – dotted line corresponds to the removed part of the cladding (**b**). Microscope image **(**Keyence VHX-5000**)** of the material (BSTS) deposited onto a 1.56 µm side-polished fiber (**c**).

Saturable absorbers were prepared both with BTS and BSTS crystals. The layer of a material was exfoliated from a bulk single-crystal using scotch tape and it was subsequently deposited onto two side-polished polarization maintaining fibers designed for single-mode operation at 1 µm or 1.56 µm (Evanescent Optics, 953 P). The fibers were embedded in a glass structure (quartz block) and subsequently polished within a few micrometers to the core in order to enable an access to evanescent wave propagating in the fiber cladding. Both fibers, for 1 μm and 1.56 μm operation, were polished perpendicularly to the slow axis hence one of the stress rods along the cut is removed. Clean fibers exhibits lossless flat transmission profile before the deposition of the material. The deposited material is attached to the polished surface due to the adhesion force. No additional polymers were used to cover and stabilize the deposited layer. The thickness of the layers deposited on side-polished fibers amounted to a few μm, for both BTS and BSTS-based devices. The geometry of the fabricated device as well as the microscope image after the deposition are depicted in Fig. 3.

When a side-polished fiber is covered with absorbing material it exhibits polarization-dependent losses^35^ due to the unsymmetrical design and it might work as a polarizer^36^. In that case the pulse generation can be caused by both nonlinear polarization rotation and saturable absorption in the material^18, 37^. It is possible to use a polarization-maintaining fiber to avoid ambiguity^21, 28^. Though the fiber was polished parallel to its fast axis, it naturally supports both TE (collinear with fast axis) and TM (collinear with slow axis) polarizations. However, as previously demonstrated^28^, the interaction of the evanescent field with the deposited material vary between these two configurations, with the TM propagation being stronger. We investigated the dependence of the polarization mode on transmission of the 1.56 µm component. The attenuation for light polarized linearly along the slow axis (TM mode) was measured to be 5.5 dB. In case of the polarization parallel to the fast axis (TE mode) the insertion loss was 3.0 dB. Consequently, in designed laser cavities all fiber components were fast axis-blocked in order to exploit the highest magnitude of the interaction. Moreover, it is well known that a non-polarization maintaining side-polished fiber with an overlay can introduce sinusoidal spectral filtering due to its birefringence^37, 38^. However, this should not be the case in described PM fiber components, as only single polarization state is transmitted (considering fast axis blocking of the employed cavity components). In order to verify that, we investigated spectral dependence of linear transmission after the deposition of BSTS. In both cases fiber components exhibited a flat transmission profile (see Supplementary Information).

**Figure 4:**
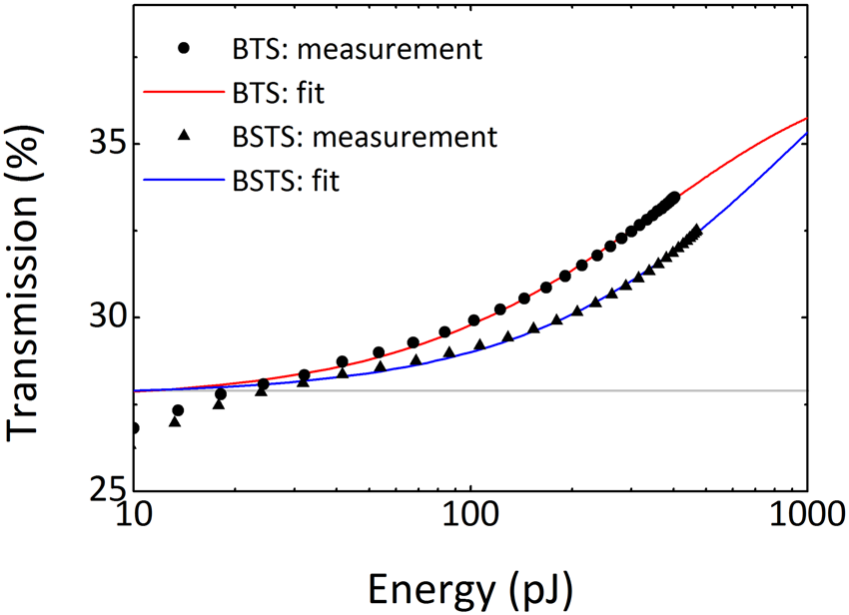
Nonlinear optical properties of BTS and BSTS saturable absorber measured at 1.56 μm.

Nonlinear optical properties of the saturable absorber prepared with the side-polished fiber designed for 1.56 μm operation are presented in Fig. 4. At first, we note that there is no saturable absorption in clean side-polished fibers. Next, both BTS and BSTS-based saturable absorbers were investigated. The measurement data points were fitted with standard slow saturable absorber model^39^, as topological insulators were previously reported to have the relaxation time in the picosecond time scale^40^ and the measurement was performed with 250-fs pulse. In the case of BTS saturable the effective measured modulation depth was equal to 5.9%. However, the saturable absorber was not fully saturated due to the limited power of pump laser available in the experiment. The simulated curve obtained after fitting experimental data is characterized by 10.1% modulation depth, 198 pJ saturation energy and 62.3% of non-saturable losses. The measurement of Sn-doped saturable absorber revealed the effective measured modulation of 4.7%, which was obtained after fitting the curve characterized by modulation depth of 13.4%, saturation energy equal to 506 pJ, and non-saturable losses at the level of 58.8%. As concluded from simulated curves, BSTS offers higher modulation depth at the same level of linear transmission, but this has to be further confirmed in the experiment with higher energy pulses. Nevertheless, in both cases the saturable absorption parameters might be considered as similar. In near-infrared wavelength range (here corresponding to ≈0.8 eV) the saturable absorption is mostly attributed to the bulk states. High modulation depth of the device together with high damage threshold makes it very suitable for the application as a modulator in passively Q-switched lasers. The same measurement has been performed for the 1 µm absorber and it can be found in Supplementary Materials section.

The saturable absorbers, designed for 1.56 μm and 1 μm were used for the generation of high-energy Q-switched pulses in erbium and ytterbium-doped fiber lasers.

### Erbium-doped Q-switched fiber laser

**Figure 5:**
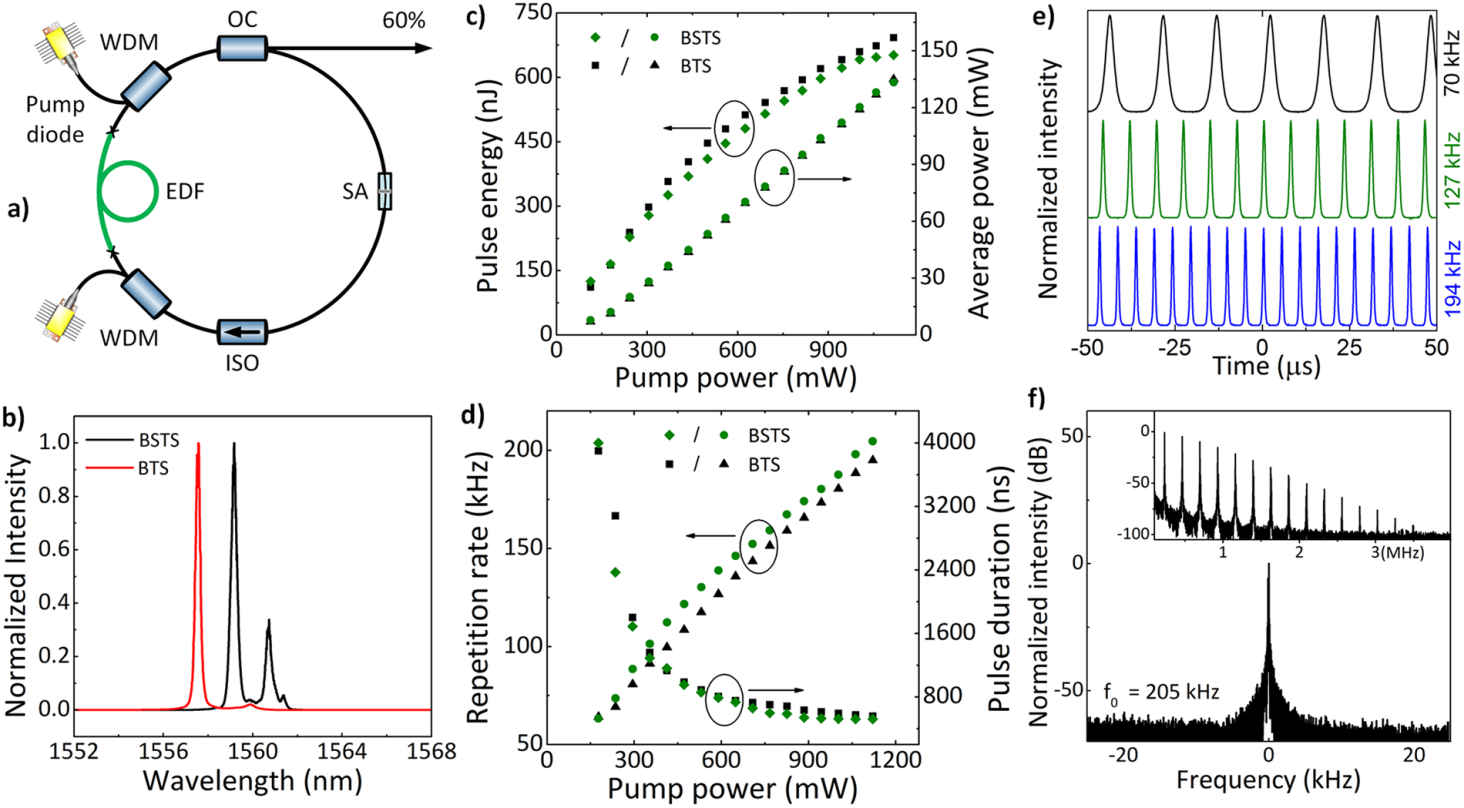
Experimental setup of the erbium-doped fiber laser (**a**). Output characteristics of the fiber laser: optical spectrum for the highest output power (**b**). Average output power and pulse energy as a function of pump power (**c**). Repetition rate and pulse width as a function of pump power (**d**). Recorded exemplary pulse trains (**e**; for BSTS saturable absorber). Fundamental beat note of radio frequency (RF) spectrum, RBW: 10 Hz. Inset: RF spectrum over the 4 MHz span, RBW: 1 kHz (**f**; for BSTS saturable absorber).

The experimental setup of the laser designed in monolithic all-fiber configuration is presented in Fig. 5(a). All fibers used in the experiment are polarization-maintaining (Panda type) and all components are fast-axis blocked, which ensures that propagating beam is linearly polarized along the slow axis. Additionally, the laser operation is immune to external factors, like mechanical vibrations. The cavity consists of 72 cm long erbium-doped fiber (nLight LIEKKI® Er80-4/125) as a gain medium. The remaining part is composed of passive single-mode fibers. The active fiber is pumped from both sides by two 980 nm semiconductor laser diodes via filter-type wavelength-division multiplexers (WDM). The combined power of both diodes reaches 1.1 W (as measured after the WDMs). The isolator (ISO) ensures a unidirectional, clockwise beam propagation. The part of oscillating light is extracted by 60% output coupler.

First we investigated the laser with BSTS-based saturable absorber. After splicing the saturable absorber the laser provides a self-start, turn-key PQS operation at any value of combined pump power between 110 mW and 1.1 W. The output spectrum recorded at the maximum pump power is presented in Fig. 5(b). The multiple peak operation is a commonly observed feature in Q-switched lasers^41^. Both peaks contribute to pulsed regime with the main being located at 1559.2 nm. The operation wavelength has not been shifted towards shorter wavelengths (i.e. ≈1530 nm), indicating that the losses introduced by the absorber are not excessive. The output power and energy characteristics are presented in Fig. 5(c). The average power increases together with pump power up to the maximum value of 133.3 mW. The maximum available pulse energy of 652 nJ is produced at the maximum available pump power of 1100 mW. Both repetition rate and pulse duration of produced pulses exhibit pump-power dependent features, which are typical for PQS laser, as can be seen in Fig. 5(d). Repetition rate increases linearly from 63 kHz up to 205 kHz for the maximum pump power of both diodes. Pulse duration decreases to a minimum value of 520 ns. Oscilloscope pulse trains recorded at various exemplary repetition rates are presented in Fig. 5(e). The laser was characterized by excellent pulse amplitude stability for all pumping powers. Fundamental beat note of radio frequency (RF) spectrum in 100 kHz span is presented in Fig. 5(f) and the spectrum in 4 MHz span is shown in the inset. The measurement was performed for the highest output power at 205 kHz repetition rate. The signal to noise ratio (SNR) was measured to be around 35 dB. Broad spectrum of harmonics indicated a stable operation. The operation characteristics of the laser Q-switched by BTS-based saturable absorber are very similar, which is presented in corresponding graphs in Fig. 5. The maximum average output power and pulse energy were 135 mW and 692 nJ, respectively. Repetition rate was adjustable from 64 to 195 kHz. The shortest obtained pulse duration was 555 ns. It is worth emphasizing that no damage to the saturable absorber was observed even at the highest intracavity energy exceeding 1 μJ.

### Ytterbium-doped Q-switched fiber laser

**Figure 6:**
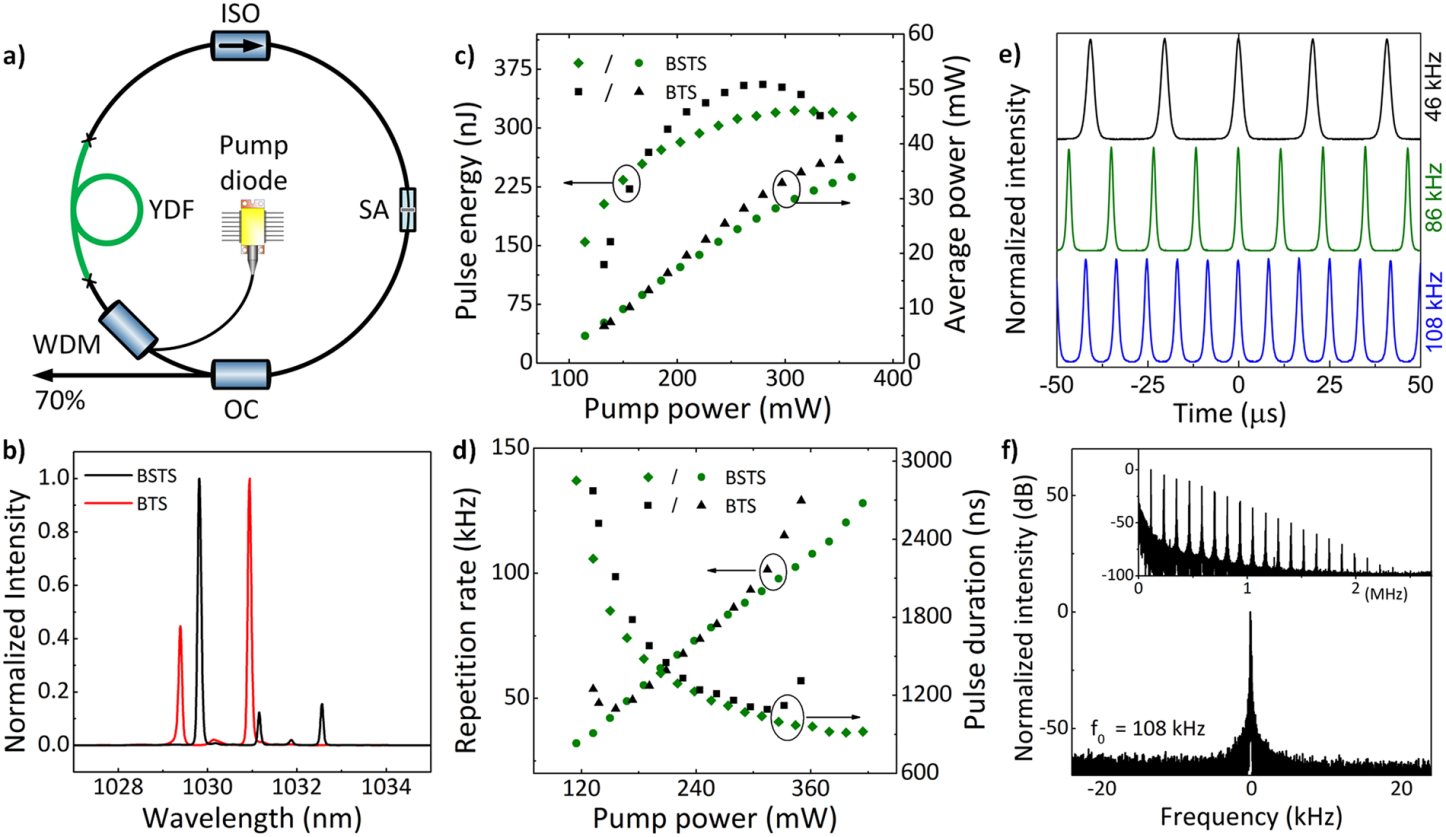
Experimental setup of the ytterbium-doped fiber laser (**a**). Output characteristics of the fiber laser: optical spectrum for the highest output power (**b**). Average output power and pulse energy as a function of pump power (**c**). Repetition rate and pulse width as a function of pump power (**d**). Recorded exemplary pulse trains (**e**; for BSTS saturable absorber). Fundamental beat note of radio frequency (RF) spectrum, RBW: 10 Hz. Inset: RF spectrum over the 2.5 MHz span, RBW: 1 kHz (**f**; for BSTS saturable absorber).

Similar to the Er-doped setup the Yb-doped laser has a simple ring geometry (Fig. 6(a)) and all of its components maintain polarization (single-mode Panda fibers with fast axis blocked). Linearly polarized 979 nm single-mode laser diode is used as a pump source and can deliver up to 400 mW. The pump light is coupled-in to the cavity via the WDM and it is subsequently absorbed in the Yb-doped fiber which acts as a gain medium. The length of the active fiber (PM-YSF-HI, Nufern), its core absorption and mode-field diameter at signal wavelength are 120 cm, 250 dB/m and 7.5 µm, respectively. The gain medium is followed by a fiber isolator which determines the directionality of the generated radiation – in this experiment a co-propagation scheme was chosen. Previously described side-polished fiber, which acts as a saturable absorber is implemented between the isolator and an output coupler which extracts 70% of the circulating radiation and transmits the remaining 30% back to the cavity through the WDM.

When BSTS-based saturable absorber is spliced in the cavity, the laser starts to operate in continuous wave (CW) regime at the threshold pump power of 58 mW (all given pump power values correspond to optical power after WDM). After increasing the pump power to 115 mW we observed transition to Q-switching. Pulsed operation could be sustained up to 362 mW of the pump power when the amplitude stability started to decrease until the laser turned to CW regime again. The spectrum of the output radiation for the highest output power is shown in Fig. 6(b). The average output power increased linearly from 5 mW up to 33.9 mW. Nevertheless, due to simultaneous increase of the repetition rate the maximum single pulse energy of 322 nJ was obtained for intermediate pump power value of 309 mW (with corresponding *f*_*rep*_ = 93 kHz and *τ* = 1.04 µs). Regardless of relatively high pulse energy the absorber was not damaged, even at the highest intracavity pulse energy of 460 nJ. The dependence of the average output power and the pulse energy versus the pump power is presented in Fig. 6(c). At the lower Q-switching threshold the pulse duration and repetition frequency were determined to be *τ = *2.85 µs and *f*_*rep*_ = 32 kHz, respectively. With an increase of pump power we observed typical changes in temporal characteristics of the output radiation. At the maximum pump power the repetition rate increased up to 108 kHz and the pulse duration was the shortest with 960 ns. The entire temporal behavior is depicted in Fig. 6(d). Furthermore, in Fig. 6(e) we present typical pulse trains at various pulse repetition rates including the lowest, one intermediate and the highest values. The recorded traces were characterized by a very good amplitude stability in the entire range of aforementioned pump power. Figure 6(f) depicts the fundamental beat note of RF spectrum (with 40 kHz span and 10 Hz resolution bandwidth) and broad spectrum of its harmonics (inset, span 2 MHz, RBW 1 kHz) for the highest output power. The signal to noise ratio of the fundamental beat note was determined to be 25 dB. The laser Q-switched by BTS-based saturable absorber offered a very similar performance, when compared to BSTS case. Both sets of data are summarized in Fig. 6. The highest available pulse energy was at the level of 356 nJ, average output power was 37 mW. The repetition rate could be controlled between 45 kHz and 129 kHz. The shortest noted pulse duration was 1.09 μs.

### Polarization state of output beam

**Figure 7:**
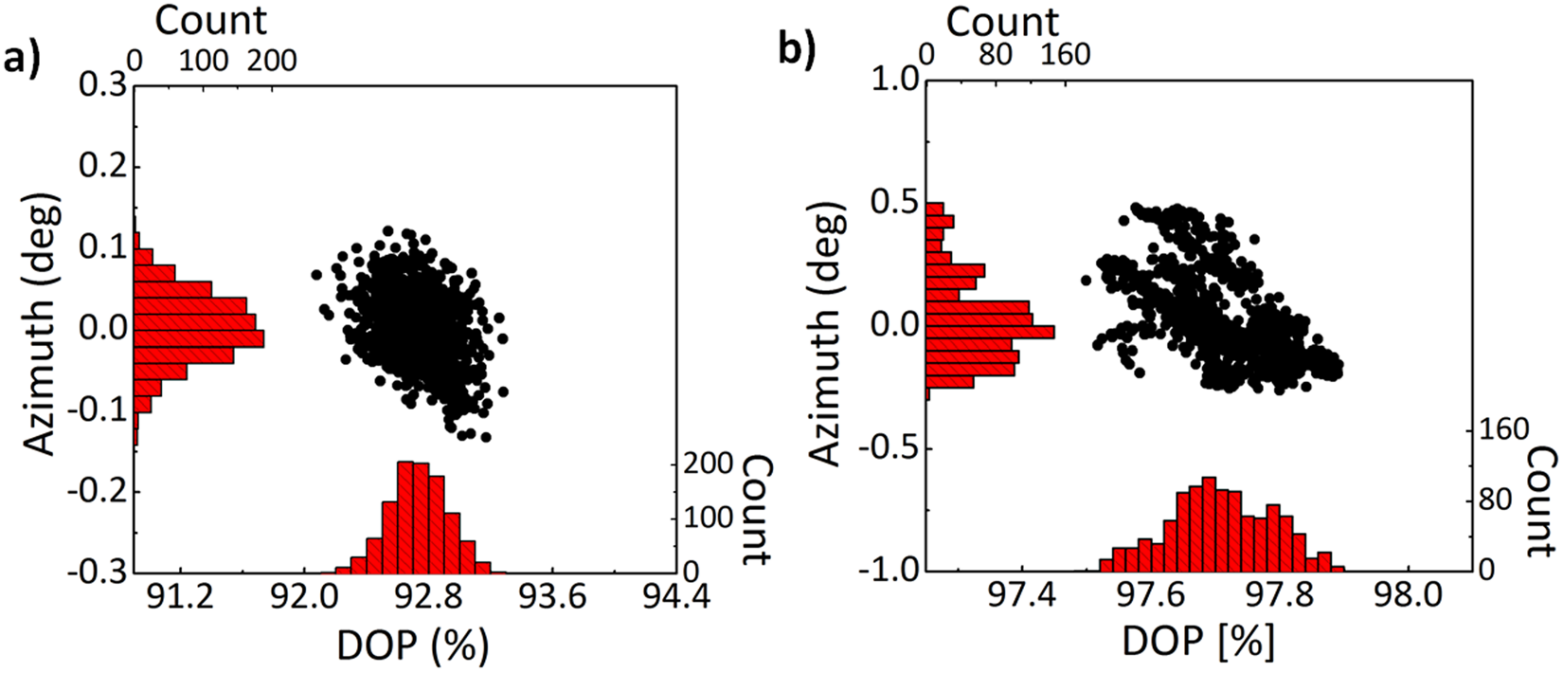
Polarization properties of output beam: azimuth angle vs. degree of polarization (DOP) measured over 1024 data points for erbium-doped (**a**) and ytterbium-doped (**b**) laser setup.

The polarization state of output beam was analyzed in order to confirm if it is linear as expected. Figure 7 presents degree of polarization (DOP) vs. azimuth angle of polarization for 1024 points measured over a period of 60 s for both laser setups. Both DOP and azimuth are also depicted in the form of histograms^42^. In the case of laser operating at 1.56 μm the mean value of DOP is 92.7% with standard deviation of 0.19%. The azimuth of polarization is characterized by mean value of 0 deg and the standard deviation of 0.04 deg. The output beam of laser operating at 1 μm is characterized by DOP of 97.7% with standard deviation of (0.08%). The azimuth of polarization is 0.03 deg and the standard deviation is 1.17 deg. Thereupon, both lasers produce linearly polarized pulses with very good polarization stability.

## Discussion and Conclusions

In Table 1 we compared current results reported in the literature with the ones presented in this work. The table includes passively Q-switched fiber lasers with nanomaterial-based saturable absorbers. The summary is limited to environmentally stable lasers based on polarization maintaining fibers, which produce linearly polarized output beam. Lasers in this configuration were demonstrated using various kinds of materials, including graphene^43^ operating around 1 µm and its derivative, reduced graphene oxide (rGO), operating at 1.56 µm^44^. The use of topological insulators was also demonstrated: both Bi_2_Te_3_ and Sb_2_Te_3_ were applied. The best performance was noted in Yb-doped fiber laser with graphene and Er-doped fiber laser with Sb_2_Te_3_, providing quite similar operation characteristics. Nevertheless, the results reported in this work are significantly better in terms of repetition rate, pulse duration, output power and pulse energy, making it much more suitable for a variety of practical applications. Those results were facilitated by high damage threshold of BTS and BSTS materials. To the best of our knowledge, there has been only one prior demonstration of the PM ytterbium-doped fiber laser Q-switched with nanomaterial-based saturable absorber^43^.

**Table 1:** Comparison of monolithic, all-polarization maintaining PQS fiber lasers based on nanomaterial saturable absorbers reported so far.

SA	Wavelength	Repetition rate	Pulse energy	Output power	Pulse duration	Ref.
**Erbium-doped PQS fiber lasers**						
Bi_2_Te_3_	1558.1 nm	26.6–47.1 kHz	17.8 nJ	0.83 mW	3.84–1.58 μs	42
Sb_2_Te_3_	1559 nm	42.0–132.0 kHz	152 nJ	18.1 mW	5.24–0.93 μs	28
rGO	1564 nm	104.0–116.0 kHz	125 nJ	14.6 mW	3.85–1.85 μs	44
BTS	1557.6 nm	64.1–195.0 kHz	692 nJ	135.0 mW	4–0.55 μs	This work
BSTS	1559.2 nm	63.2–204.6 kHz	652 nJ	133.3 mW	4–0.52 μs	This work
**Ytterbium-doped PQS fiber lasers**						
Graphene	1027 nm	28.9–110.0 kHz	141.8 nJ	15.6 mW	3.2–1.3 μs	43
BTS	1030.9 nm	45–129 kHz	356 nJ	37.0 mW	2.77–1.09 μs	This work
BSTS	1029.8 nm	32–108 kHz	322 nJ	33.9 mW	2.85–0.96 μs	This work

In conclusion, we have demonstrated for the first time that Bi_2_Te_2_Se and Sn-doped Bi_2_Te_2_Se can be used as a broadband saturable absorber. Thin layer of the material was mechanically exfoliated from bulk crystals and subsequently deposited onto a side-polished polarization-maintaining fibers enabling interaction with the evanescent field of the propagating beam. Nonlinear optical properties of the fabricated device have been investigated and the absorbers have been employed for pulse generation at 1 µm and 1.56 µm spectral ranges. We have successfully obtained passive Q-switching regime in both polarization-maintaining ytterbium- and erbium-doped fiber resonators. The absorbers exhibited high damage threshold, while the lasers were insensible to various disturbing factors like mechanical vibrations. Both lasers are characterized by the best performance observed in all-polarization maintaining Q-switched oscillators based on novel saturable absorbers.

## Methods

### Crystal growth

Single crystal of Bi_2_Te_2_Se (BTS) and Sn-doped Bi_1.99_Sn_0.01_Te_2_Se (BSTS) were grown by modified Bridgman method^34^ in quartz ampules sealed under high vacuum ≈10^−6^ Torr. For synthesis stoichiometric amounts of Bi, Te, Se and Sn elements with 5 N – 6 N purity were used. Obtained crystals were easily cleaved along the (001) crystallographic plane.

### SEM measurements

SEM analysis was done using Zeiss Auriga field emission (Schottky type) scanning electron microscope (FESEM), operating at 15 kV incident energy.

### XRD measurements

Crystal structure and crystal quality was verified by X-ray powder diffraction using Siemens D500 diffractometer equipped with a semiconductor, high-resolution Si:Li detector. The characteristic peak position was indicated from ICDD PDF-2 29–0247 database.

### Hall and resistivity measurements

The Hall measurements were performed using transport option of the Physical Property Measurement System of Quantum Design in temperature range 2–300 K. For Hall and resistivity measurements standard four point probe method was used.

### Nonlinear transmission measurements

Nonlinear optical properties of the saturable absorber were investigated using all-fiber balanced twin detector method, as described in^45^, pumped by 250-fs, 100-MHz repetition rate fiber laser operating at 1.56 µm. This experiment was performed with linearly polarized light along the slow axis of the fiber to resemble the experimental conditions of laser’s operation.

### Laser performance measurement equipment

Laser performance was monitored with optical spectrum analyzer (Yokogawa AQ6370C), radio frequency analyzer (Agilent Technologies N9010A), 350 MHz digital oscilloscope (Agilent DSOX3034 A), both connected to 5 GHz fast photodiode (Thorlabs DET08CFC), and a power meter. A polarimeter (Thorlabs PAX7510IR1-T) was used to analyze polarization state of output beam.

### Data Availability

The datasets generated during and/or analysed during the current study are available from the corresponding author on reasonable request.

## Electronic supplementary material

Supplementary information
