# Supplementary material for: Exploiting nonlinear properties of pure and Sn-doped Bi2Te2Se for passive Q-switching of all-polarization maintaining ytterbium- and erbium-doped fiber lasers
Source: Sci Rep. 2017 Aug 7;7:7428. doi: 10.1038/s41598-017-07706-7 (PMC5547047; doi:10.1038/s41598-017-07706-7)
Supplement: Supplementary file 1 — Supplementary information [file 41598_2017_7706_MOESM1_ESM.doc]

**Supplementary information**

**Exploiting nonlinear properties of pure and Sn-doped Bi_2_Te_2_Se for passive Q‑switching of all-polarization maintaining ytterbium- and erbium-doped fiber lasers**

**Jakub Bogusławski^1^,**^†^**, Maciej Kowalczyk^1^,**^†,*^**, Przemysław Iwanowski^2,3^, Andrzej Hruban^2^,
Ryszard Diduszko^2,4^, Kazimierz Piotrowski^2^, Krzysztof Dybko^2^, Tomasz Wojciechowski^2,3^, Marta Aleszkiewicz^2^, & Jarosław Sotor^1^**

*1. Laser & Fiber Electronics Group, Faculty of Electronics, Wrocław University of Science and Technology,
Wybrzeże S. Wyspiańskiego 27, 50-370 Wrocław, Poland*

*2. Institute of Physics, Polish Academy of Sciences, Aleja Lotnikow 32/46, PL-02-668 Warsaw, Poland*

*3. International Research Centre MagTop, Aleja Lotników 32/46, PL-02-668 Warsaw, Poland*

*4. Tele and Radio Research Institute, Ratuszowa 11, PL-03-450 Warsaw, Poland*

*† Those authors contributed equally.*

*E-mail:* [*m.kowalczyk@pwr.edu.pl*](mailto:m.kowalczyk@pwr.edu.pl)

The quantitative point chemical composition of Bi_2_Te_2_Se (BTS) was verified using energy dispersive X‑ray spectroscopy (EDX) system QUANTAX 400 Bruker coupled with the Zeiss Auriga field emission (Schottky type) scanning electron microscope (FESEM), operating at 15 kV incident energy. The analyses was performed at the middle area of the crystal ingot and it was confirmed as Bi_2_Te_2_Se. The results are presented in Fig. 1. The amount of Sn in Sn-doped BTS crystal was too small to reliably verify it with this method.


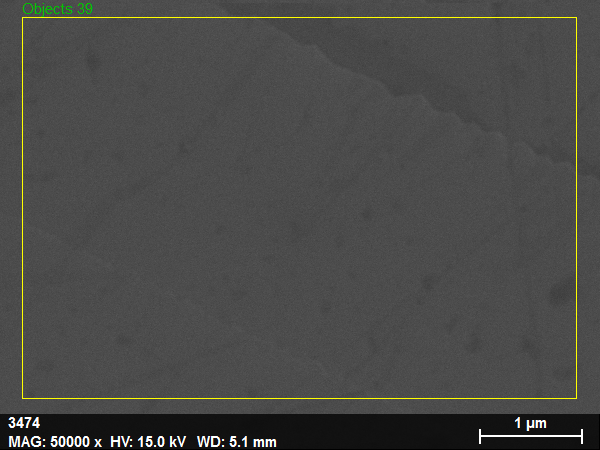


**Fig.1.** SEM image of BTS crystal with marked area of analysis (a) and obtained EDX spectrum (b).

The morphology of the surface of freshly cleaved BTS and BSTS crystals was investigated by the atomic force microscopy (AFM) using a Nanoscope MultiMode system at ambient conditions. The images were taken in dynamic Tapping mode using silicon nitride cantilevers. Flat terraces of micrometric width have the height of multiple 1 nm which corresponds to the height of one quintuple set of layers. The results for pure and Sn-doped BTS crystals are shown in Fig. 2 and Fig. 3, respectively. The ~1 nm terraces corresponds to the single quintuple, which indicated that the sample was cleaved along the plane of cleavage. The measurement also confirms flatness of cleaved samples in the micrometers scale, which enables the excellent contact with side-polished fiber surface.


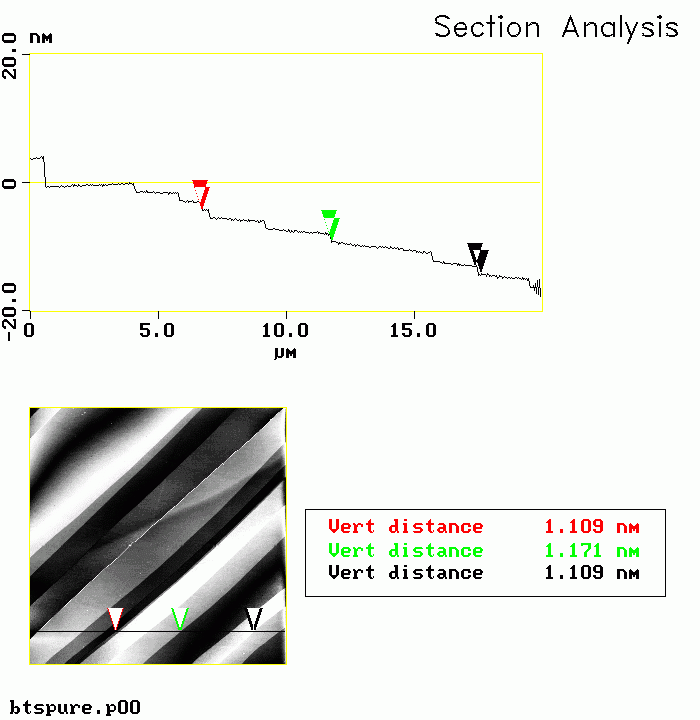


**Fig.2.** AFM analysis of pure BTS.


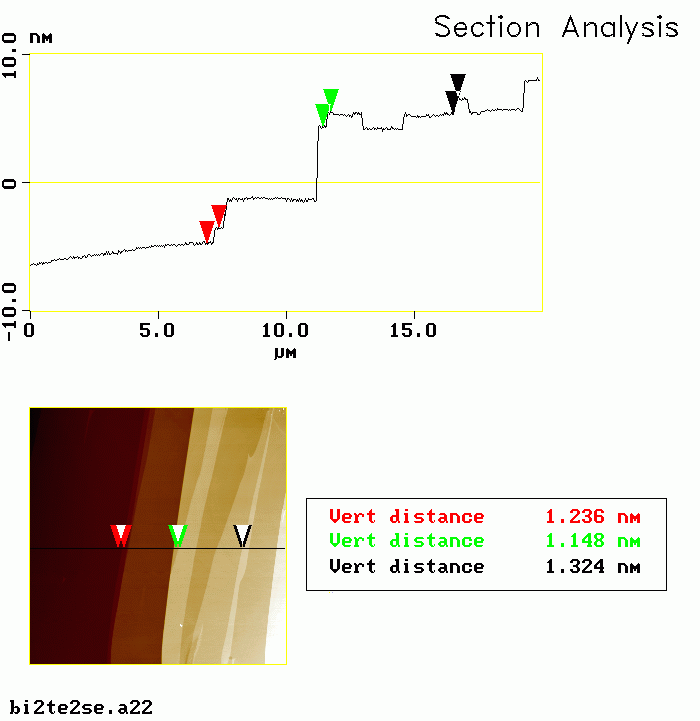


**Fig.3.** AFM analysis of Sn-doped BTS.

We have investigated the spectral transmission of the fabricated saturable absorbers around 1 µm and 1.56 µm. The graphs shown in Fig. 1 demonstrate flat transmission profile without any filtering features. The measurements were performed with Yb-based amplified spontaneous emission (ASE) source (Fig. 1(a)) and white light source (Yokogawa AQ4305, Fig. 1(b)).


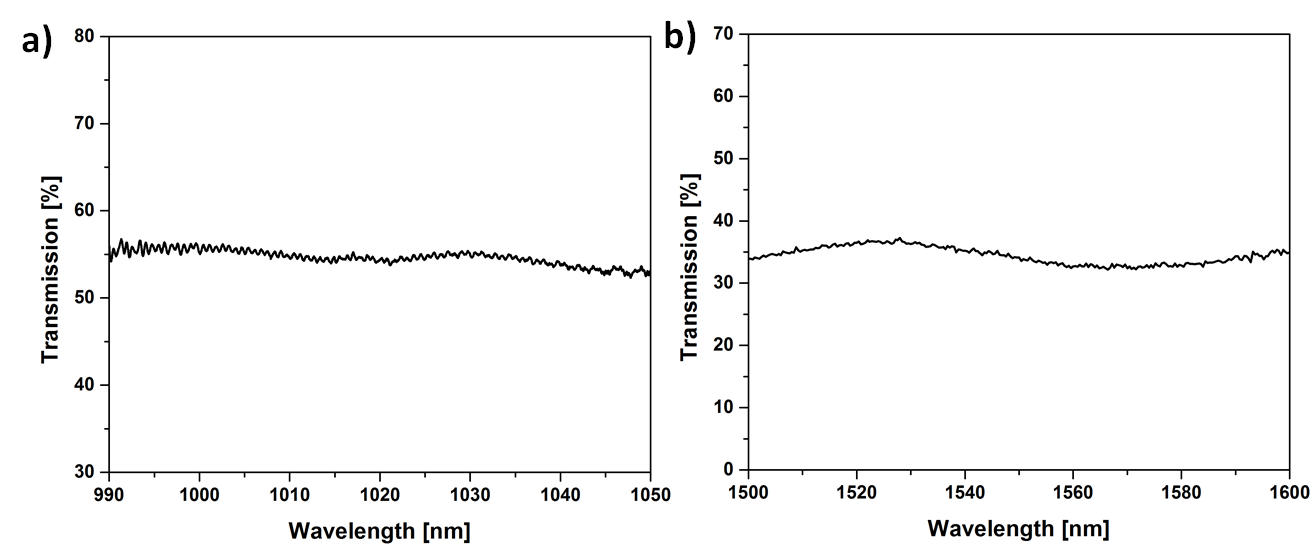


**Fig.4.** Spectral transmission of the fabricated BSTS saturable absorber device around 1 µm (a) and 1.56 µm (b).

The saturable absorbed for the application in ytterbium-doped fiber laser was prepared in the same way as described for erbium-doped fiber laser. A layer of material was exfoliated using scotch tape from bulk single crystal of BTS (BSTS respectively) and deposited on polarization-maintaining side-polished fiber designed for single mode operation at 1 µm spectral range. We investigated the nonlinear optical properties of the fabricated absorber with the standard fiber based saturable absorption measurement setup^1^. A solid-state Yb:KGW laser delivering 200 fs pulses at 62 MHz repetition was employed as a light source^2^. After coupling the beam into the fiber setup 50 mW of average optical power was available. Due to self-phase modulation and fiber dispersion, the pulses acquire chirp within the fiber. Consequently, the pulse duration at the absorber amounts to 8.5 ps (measured with APE PulseCheck autocorrelator). The results for BSTS-based saturable absorber measurement are presented in Fig. 5. The measurement revealed 11.5% modulation depth and 36.8% of non-saturable losses. However, the saturable absorber was not fully saturated due to the limited energy of laser available for the experiment. The experimental data was fitted slow saturable absorber model^3^ which indicates possible modulation depth up to 21.6%, non-saturable losses of 26.7% and saturation energy of 400 pJ.


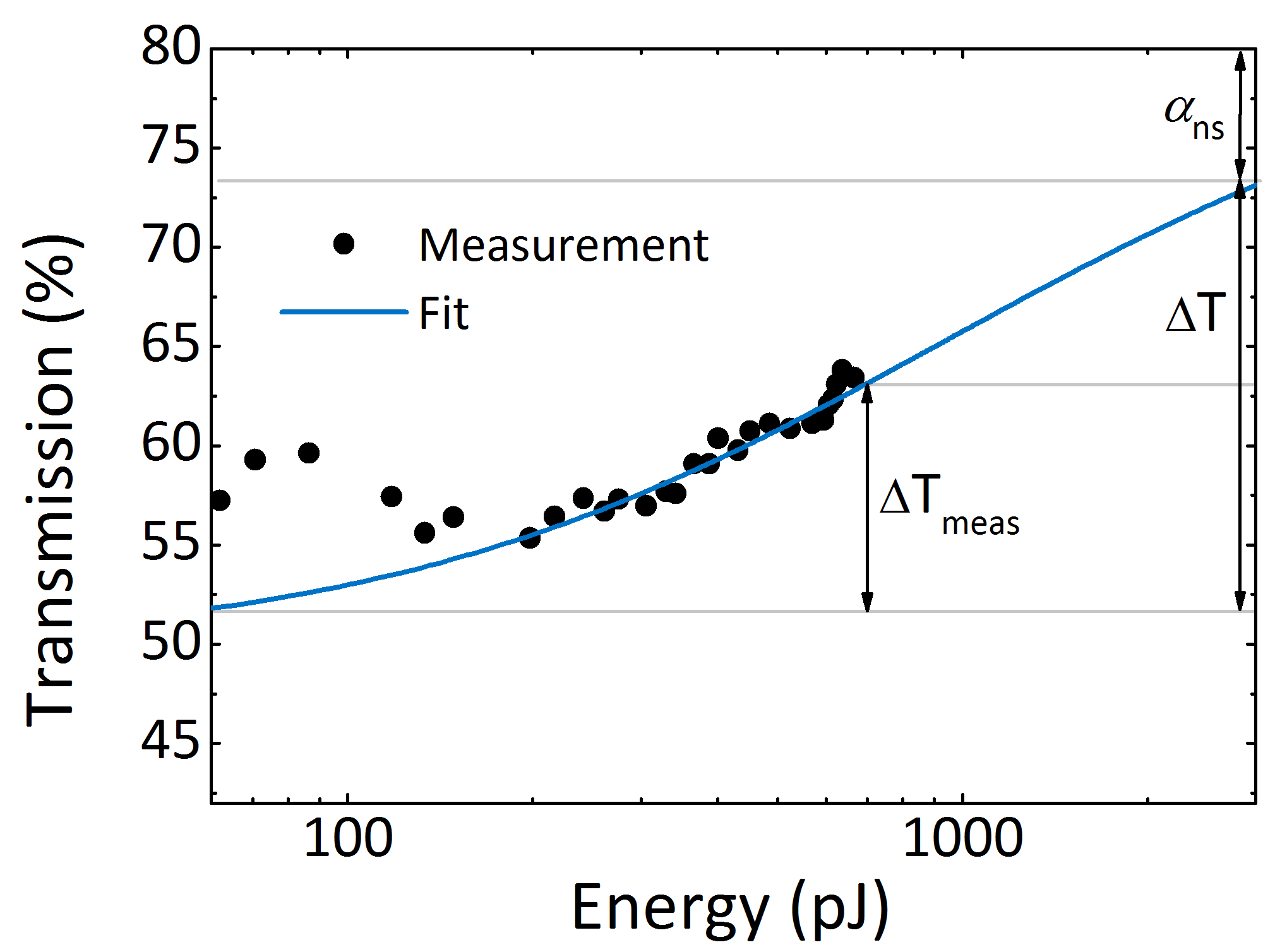


**Fig.5.** Nonlinear transmission of BSTS-based saturable absorber measured at 1 μm wavelength.

1. Sobon, G. Mode-locking of fiber lasers using novel two-dimensional nanomaterials: graphene and topological insulators [Invited]. *Photonics Res.* **3,** A56 (2015).

2. Kowalczyk, M. *et al.* 59 fs mode-locked Yb:KGW oscillator pumped by a single-mode laser diode. *Laser Phys. Lett.* **13,** 35801 (2016).

3. Grange, R. *et al.* New regime of inverse saturable absorption for self-stabilizing passively mode-locked lasers. *Appl. Phys. B* **80,** 151–158 (2005).
